# Exploring varieties of knowledge in safe work practices - an ethnographic study of surgical teams

**DOI:** 10.1186/1754-9493-5-21

**Published:** 2011-09-13

**Authors:** Sindre Høyland, Karina Aase, Jan Gustav Hollund

**Affiliations:** 1 Department of Health Studies, Faculty of Social Sciences, University of Stavanger, Stavanger, Norway; 2 Department of Anesthesia, Stavanger University Hospital, Stavanger, Norway

**Keywords:** Safe work practices, knowledge, interdisciplinary team work, surgical operations, Norway

## Abstract

**Background:**

Within existing research in health and medicine, the nature of knowledge on how teams conduct safe work practices has yet to be properly explored.

**Methods:**

We address this concern by exploring the varieties in which knowledge is expressed during interdisciplinary surgical operations. Specifically, the study was conducted in a surgical section of a Norwegian regional general hospital, between January and April of 2010, by means of an ethnographic design combining detailed non-participant observations, conversations and semi-structured interviews.

**Results:**

Based on an analysis of the gathered data, we identify three particular themes in how knowledge is expressed by operating room personnel: (i) the ability and variety individuals demonstrate in handling multiple sources of information, before reaching a particular decision, (ii) the variety of ways awareness or anticipation of future events is expressed, and (iii) the different ways sudden and unexpected situations are handled by the individual team members.

**Conclusions:**

We conclude that these facets of knowledge bring different insights into how safe work practices are achieved at an individual and team level in surgical operations, thus adding to the existing understanding of the nature of knowledge in safe work practices in surgical operations. Future research should focus on exploring and documenting the relationships between various elements of knowledge and safe work practices, in different surgical settings and countries.

## Background

Traditionally, the process of ensuring clinical competency have been subjected to what Schön [1] terms a "technical rationality", that is a state of mind or mental model of problem solving using established scientific theories and techniques. However, health care literature in recent years have also looked to specific safety principles used in high reliability sectors [2-5], and recognized that the individual technical skills are only one part of the total skill repertoire applied by individuals as part of a team. Despite this, the dominance of the technical rationality seems to prevail, much of which can be attributed to weaknesses in the identification, understanding and training of health care specific team skills [6,7], in the commitment of resources and time necessary to ensure team training [4,8], and in the overall focus on research and development of a scientifically grounded model to explore and measure the dynamics and performance of interdisciplinary teams [7]. This suggests that the nature of interdisciplinary teamwork in health care needs to be explored in ways that reveal the specific and unique characteristics of team practices in this sector. Understating the need for further explorations, Flin & Mitchell [9] suggest that there is a lack of investigation into the culture and behavior patterns of surgical working life, i.e. the operating room. Specifically, while some studies have looked into the nature of knowledge in the operating room, such as team level tacit knowledge [10], nurses' knowledge of individual surgeons [11], and nurses' selective use of gatekeeping practices [12], other aspects of knowledge in the operating room remain unexplored and consequently unidentified. Thus, the aim of this paper is to explore and document the nature of the knowledge interdisciplinary teams use in surgical operations, in order to achieve safe work practices.

## Main concepts

Given the aim of documenting the nature of knowledge in safe work practices, an understanding of both concepts should be provided.

From an evidence-based medicine (EBM) perspective, knowledge rests on the model of technical rationality, where an individual practices problem solving according to established scientific theories and techniques [[1], p. 21]. The EBM-perspective's dominance in medicine has resulted in a strong focus on the creation, storage and distribution of codified/explicit 'text-book' knowledge [13], expressed as procedures, protocols, routines, etc. However, many researchers believe that one must also account for other kinds of knowledge health care personnel use in practice, such as clinical judgment and expertise [14-19]. Greenhalgh et al [20] support this view, stating that: "...multidisciplinary teams balance encoded knowledge, in the form of standardised outcome measurement, with tacit knowledge, in the form of intuitive judgement, clinical experience and expertise, in the process of clinical decision making" (p. 183). Thus, in this paper we define knowledge as comprised of explicit/encoded aspects shaped by text-book understandings of various procedures, and as comprised of tacit aspects shaped through experience and exposure to various clinical situations.

In understanding the concept of safe work practices, we focus on the connection between the concept's basic components; safety and practice. Within research in health and medicine, a first connection between safety and practice appears in the *safety-driven *focus on identifying and training individual and team-based skills, aimed at improving *clinical and surgical practice *[21-24]. The concept "Community of Practice" (CoP) represents a second connection between safety and practice. Specifically, a CoP can be viewed as a network of people who share information, build on existing knowledge, and develop expertise to solve problems for a common purpose [25,26]. One such purpose is the pursuit of evidence to support current practices [25], including the improvement of skills, outcomes and consequently safety. Thus, the individual's and team's ability to conduct and complete operations with a minimum of complications - that is safe work practices - can be understood as a product of the measures aimed at improving the skills, knowledge and/or expertise levels of individuals and teams.

## Methods

This paper presents the results from a qualitative study. The goal of qualitative research is to gather an in-depth understanding of human behavior and the reasons that governs such behavior, or as Larsson [27] states: "The aim of [qualitative] research is not to confirm or refute hypothesizes by using statistical methods, but to increase our understanding of complex human or social phenomena by discovering patterns of human thinking and acting. Anesthesiologists at work is one example of humans in action" (p. 444).

More specifically, within a qualitative research tradition, the study presented in this paper applies an ethnographic approach [28], combining detailed non-participant observations, conversations and semi-structured interviews. By ethnography we imply "a general approach to the exploration and understanding of social settings and social processes" [[29], p. 228]. The main benefit of ethnography is that it enables the researcher to "...enter into close and relatively long-term contact with people in their everyday life" [[30], p. 66].

### Ethical concerns

The study was conducted in a surgical unit of a Norwegian regional general hospital. Based on the approval and recommendations of the Norwegian Social Science Data Services (NSD), all potential participants of the study (sample) were informed via presentations prior to the field work. During these presentations, participants were handed a written information form that included information on the aim of the study and anonymity issues, and also a field for signing informed consent. Observations were only conducted when every member of the operating team had agreed to be observed. In situations where information had not been given and/or consent not obtained beforehand, this was taken care of before the operation began.

### Sample

A typical operating team consists of 1-2 operators (surgeons), 2 operating room nurses, 1-2 nurse anesthetists, and 1 anesthetist physician. Table 1 illustrates the groups observed, the total sample size, the numbers who gave their informed consent, the numbers who were actually observed, and the numbers who were interviewed. The interviews lasted an average of 43 minutes. It is relevant to note that the overall composition of the operating teams varied constantly from one operation to the next (ad hoc), also documented in other studies [31]. Thus, the total of 27 observed operations also represent the number of observed team variations.

**Table 1:** Distribution of observations and interviews

Groups observed	Sample	Informed	Observed	Interviewed
Anesthetist physician	9	5	5	2

Nurse anesthetist	15	14	11	3

Operating room nurse	22	15	15	2

Operator (surgeon)	45	16	16	4

Manager (interviews)	NA	NA	NA	4

Total (% of sample)	91	50 (55)	47 (52)	15 (16)

Interviews and observations were sampled to cover variety. In interviews this was achieved by ensuring variety across different types of professions, as shown in table 1. Variety was also achieved through age groups (33-54 years, 43.9 years on average), sexes (5 females, 10 males), and levels of experience as a specialist (2-36 years, 12.6 years on average). In terms of the observations, variety was achieved within the two main categories of elective (planned) and immediate (within 72 hours) surgery, and by attending different types of operations within the main categories, as listed in table 2.

**Table 2:** Distribution of observation type and duration

Type of observations	Elective	Immediate	Total/Hours
Variants of fracture	1 (00:45)	11 (21:50)	12 (22:35)

Variants of revision	2 (03:30)	1 (02:00)	3 (05:30)

Achilles extension	3 (06:30)	NA	3 (06:30)

Back stabilization	2 (12:30)	NA	2 (12:30)

Other	7 (15:20)	NA	7 (15:20)

Total/Hours	15 (38:35)	12 (23:50)	27 (62:25)

### Practical methodology

At the beginning of each observation, the operation was numbered (1-n) and specified in terms of type of operation and participants. A principal researcher (SH) and a co-researcher (JGH) were present at the majority of the operations, to ensure comparison and internal validity of the observations. Validity can be understood as the researcher's ability to interpret observations that corresponds accurately to the real world. Hereunder, internal validity refers to "the extent to which scientific observations and measurements are authentic representations of some reality" [[32], p. 32], implying that the comparison of observations between two or more researchers will strengthen this type of validity. Furthermore, transcriptions were done individually, and focused on identifying emergent themes. This was followed by comparison of transcriptions and themes between observers, by means of discussions, to confirm, adjust or dismiss the understandings. To further strengthen the correspondence between the observations made by the researchers and the real world (validity), validation via respondents (respondent validation) also occurred during conversations and interviews.

In terms of the interviews, the main priority was to achieve a working synergy between the observations and the interviews, given our interest in respondent validity. This required that the interviews had an open nature that allowed for the inclusion of observational findings. Hence, a semi-structured interview guide was constructed, focusing on the acquisition and use of knowledge and skills, such as personal techniques, reaction to problems and critical situations, formalized training, and so forth. Both the principal researcher (SH) and the co-researcher (JGH) conducted the interviews, mainly individually but also in tandem (during 2 interviews).

Identical to the semi-structured interviews, the main purpose for initiating conversations was to approve, adjust or dismiss existing observations. A total of 35 informal conversations were conducted.

### Analysis

One aspect of the analysis process was the triangulation of findings from observations, not only via researcher comparison of notes and transcripts but also via respondent validation during interviews and conversations [33]. This triangulation helped to identify, adjust and dismiss emergent themes, and also assisted in improving the general understanding and the specific details of what was going on in the operating room. Through analytical triangulation [33], all three researchers (SH, KA, and JGH) were involved in the analysis process. Specifically, the analysis consisted of repeatedly reading the raw observational and conversational data, until the relationships between the series of events that occurred during the particular operation became clear. These events created an episode, defined as a series of related events that form a "bigger story". The episodes were then read and compared repeatedly by the researchers, individually and in tandem, until the particular emergent theme became visible in the material. A theme is defined as a clear "red line" that runs through more than one episode. Combined, the two analyzing strategies for identifying episodes and themes complemented each other. Specifically, the emphasis on episodes is supported by Nielsen's [34] story telling approach, providing *a rich and unique picture *of findings, while a focus on themes are comparable to the categorization techniques described by Miles and Huberman [35], providing *a structured and transparent picture *of findings.

## Results

The findings include episodes that demonstrate varieties and themes in how knowledge is expressed in interdisciplinary operations, as part of safe work practices. The selected episodes, derived from field notes (transcripts of notes from observations and conversations), are representative of the particular theme.

### Theme 1 - The processing of multiple sources of information - a requisite in decision making

The first identified theme in the data material is the ability and variety individuals demonstrate in handling multiple sources of information before reaching a particular decision. This is observed in the following episodes:

#### Episode 1 - "The operator's decision making"

Before starting the procedure in this particular operation, the main operator gathers his team for a briefing by a monitor displaying the patient's X-rays. During the briefing, the main operator describes the patient's condition and history, and he also explains the specific steps involved in the coming procedure (pointing and illustrating via the X-rays). He seems to be seeking approval of the procedure. At a later time in the procedure, the main operator is confronted with a choice between method A and method B. He again gathers his team by the X-rays, and receives inputs from his team and from what he sees in the pictures. The operator then makes his decision. Several X-rays are later taken, to confirm the decision.

#### Episode 2 - "Problem solving kicks in"

During preparations for this operation, uncertainty concerning the patient's position can be seen. Problem solving then kicks in: The anesthetist nurse checks the planning system Orbit for information on the pre-anesthesia assessment of the patient from the day before. She also confers with the 1^st ^operating room nurse. Neither the system nor the operating room nurse provide any clear answers. The 1^st ^operating room nurse takes over the problem solving task, and asks the 2^nd ^operating room nurse to enquire with the main operator. At last, an answer is obtained on the position of the patient.

Both episodes illustrate how information gathering from multiple sources, both technological and human in nature, enables the individual and team to reach a particular decision when confronted with uncertainty.

### Theme 2 - The anticipation of future events - a way of "being prepared"

A second theme in the data is seen from the variety of ways awareness or anticipation of future events is expressed. The following episodes display this theme:

#### Episode 1 - "Combining tacit and explicit elements"

During the preparations for this particular operation, the 1^st ^nurse anesthetist prepares the anesthesia equipment, including back-up solutions, prior to the patient's arrival. These preparations are regulated by procedures, she explains. Before the operation begins, the 1^st ^nurse anesthetist scans the patient's urinary bladder to make sure it is empty. Upon enquiry, she explains that this activity is not regulated by procedures, but a result of previous experiences from situations where too much urine accumulated in the patient's bladder. Before the operation begins, the 1^st ^operating room nurse has also prepared several alternative sets of gloves. She explains this action by the need to be prepared, since a plastic surgeon she is unfamiliar with will be present. Later in the operation, the 2^nd ^nurse anesthetist (that replaces the first due to a break) notices that the large plastic syringe with the sleeping medicament is about to be depleted, but he has prepared a new one beforehand. At the end of the operation, the 2^nd ^nurse anesthetist has already called on the patient for the upcoming operation.

#### Episode 2 - "A continuous focus on injury prevention"

During this operation, the position of the patient is checked several times and at different stages, by the anesthetist nurses, the operating room nurses and the main operator. Specifically, during preparations belts and blankets are removed from the operating bench. This, we are explained, is to prevent pressure injury when a patient remains in a given position for a prolonged period. When the main operator arrives in the operating room, he also reviews and confirms the patient's position. During the procedure, the operating room nurse massages and also lifts the arms and legs of the patient, in order to improve circulation and prevent damage. Near the end of the procedure, the operating room nurse looks under the table to check the patient's position and to make sure no injury has occurred during the operation.

Actions in both episodes are triggered either by procedures (preparing equipment, preventing damage) or experience (*continuous *focus on preventing injury, checking urine, preparing gloves and syringe, calling on patient early), thereby demonstrating that the ability to plan ahead of future events depends on a combination of both explicit and tacit knowledge elements.

Theme 2, concerning the anticipation of future events, is supported by an interview with an anesthetist physician: "It is partially a craft... the basic principles are necessary, but techniques can be adapted to achieve the same goal. For example, during a procedure where entering of a needle is involved... I use to mark the skin with the hollow end of a pen, to ensure that when a swelling occurs the mark will still be there, and I will not need to "feel" [my way to the artery] again when I enter the needle. [This is also important] when the pulse gets weak, the patient is ill, and you do not know where the artery really is." This personal technique illustrates how a procedure for entering the vein is "transformed" into a tacit ability for anticipating and handling future events of this kind, such as the patient turning ill and the vein access becoming more difficult.

### Theme 3 - The handling of the unforeseen - when it happens

A third theme in the data is displayed through the different ways sudden and unexpected situations are handled by the individual team members. Our definition of the unexpected is situations that occur infrequently during operations. The following episodes are illustrative:

#### Episode 1 - "The physician's handling of the unforeseen"

During the preparations for this particular operation, a patient associated with difficult vein access arrives. It is discovered that the patient has received no pain relieving medicaments (the "unforeseen" event). The nurse anesthetist tries to insert a needle into the patient's arm, with no luck. The same occurs when the anesthetist physician attempts to enter the patient's foot. Reflecting out loud on this information, including the difficult vein access of the patient, the physician explains that it is better to proceed inside the operating room, to gain more space and limit circulation of people. Once in, the physician attempts a few more times to enter the veins of the patient's arm, with no success. He then considers going into the groin, but rejects this alternative. Upon enquiry later, he explains that this decision was made based on the unclean state of the groin area, and also the fact that the placement of a cannula here would become uncomfortable to the patient for her scheduled stay at the hospital over several days. Following this reflection, the anesthetist physician decides to enter the neck, and uses ultrasound equipment to locate an area with potentially good veins. He then repeatedly attempts to insert needles and locate a vein in the identified area, with no success. The physician takes a step back and seems to calm down and reflect on the current situation, before he decides to make a new attempt in another area of the neck. In preparation of this task, he asks that the table is tipped over more so that the head points down (Trendelenburg Position), to improve circulation. Finally, he hits a vein.

In this episode, the anesthetist physician was able to handle the unforeseen element by building on existing information (knowledge of patient type and the failed attempts), by being aware of the current situation and equipment (moving into a less crowded room, use of ultrasound equipment), and also by considering the future consequences of his actions (rejecting insertion into the groin). The combination of all these tacit knowledge elements enabled him to handle the unforeseen situation successfully.

#### Episode 2 - "The helping hand, and calmness..."

During preparations for an operation, the team is suddenly informed that a dental hygienist is to conduct a parallel procedure, to remove tartar. This was not planned for by the team, as expressed by the main operator: "I was not informed that a dental hygienist would be present today - I first received this information in the entrance to the operating room". A conversation with the nurse anesthetist reveals the same impression: "It is terrible to get caught in the middle - it is as if you know nothing at all". However, despite individual concerns for not being informed and prepared, the team shows no signs of increased stress levels during the operation. This is seen in the general willingness to lend each other a "helping hand". For example, the main operator asks the dental hygienist whether she needs any equipment, followed by the operating room nurse assisting in obtaining the particular equipment the dentist requests. The operator also helps in positioning the operation lamp, to improve the lighting conditions for the dental hygienist.

This episode demonstrates two specific tacit knowledge elements that enabled the handling of this particular unforeseen situation: (1) The ability to remain calm, and (2) assist each other in the completion of individual tasks.

Theme 3, on the handling of the unforeseen, is also supported by an interview with an operator: "As the main operator... you apply previous experiences... if plan A does not work, it is important to know what equipment is available, [and for example] I know that the plastic surgeons have something I can borrow. If something is missing, we then know that we have the same dimensions on the screws [in another instrument shrine] to replace what we dropped on the floor." In this example, when confronted with the unexpected, the operator draws on her own experiences, the knowledge of available equipment (also external), and the ability to improvise by using similar equipment. The example also illustrates that a decision on how to proceed, given the lack of a particular piece of equipment, depends on both personal experiences with similar situations (existing information) and a knowledge of what equipment exists and/or can be improvised on (current information). The coordination of these information types supports theme 1 concerning the processing of multiple sources of information.

Next, we will discuss how our findings relate to existing health and medicine literature, to test the validity of the findings, followed by a reflection on the practical implications to surgical practices.

## Discussion

In analyzing the results presented above, a comparison can be made to the understanding of an expert within anesthesia, as described by Smith et al [36]. In their view, an expert is characterized by the ability to simultaneously balance many different sources of knowledge, such as past learning (formal and experienced) and an understanding of the dynamic situation (patient and equipment signals). This balancing is exemplified by the ability the anesthetist physician demonstrated (episode 1, theme 3) in handling an unforeseen situation (lack of anesthesia), by combining and understanding the existing information (patient type), the current situation (failed access to vein, access to ultrasound machine), and the future consequences of actions (patient information). In other words, the handling of the unforeseen through an awareness of existing information, current and past experiences and situational possibilities becomes an expression of what constitutes an expert.

In another study, Patel et al [37] identify the ability a primary care team demonstrates in distributing responsibility for a particular patient problem according to expertise. This ability allows the team to process large amounts of patient information, thereby reducing the load on the single individual. The finding by Patel et al [37] can be compared to the different ways individuals demonstrate in handling multiple sources of information in this study, before reaching a particular decision (theme 1). For example, the operator (episode 1, theme 1) handled information from multiple sources during his decision making process, but the information was clearly defined within his "zone of responsibility" (how to proceed with the operation and procedure). Many sources of information can thus be combined within each zone that, when put together, enables the team to process large amounts of information. The finding supports the understanding of distributed responsibility, as described by Patel et al [37].

Another comparison can be made between the ability to anticipate future events (theme 2), and what Friedman & Bernell [10] identifies as an ability to anticipate another team member's actions due to shared experiences. While theme 2 does not bring additional clarity to the understanding of "shared experience", the theme and related episodes suggest that the ability to anticipate is comprised of both explicit knowledge, such as procedural elements (equipment preparation, patient positioning, injury prevention), and tacit knowledge, such as unscripted elements (checking urine, preparing gloves and syringe, calling on patient early, *continuous *focus on preventing injury).

We have described the unique ways members of the operating team combine different elements of knowledge, in order to handle the unforeseen, process large amounts of information, and anticipate future events. How can this insight be transferred to and benefit actual operating room practices? We suggest that one approach is to gather all operating room staff at the particular section/department at regular weekly or monthly meetings, where experiences on combining knowledge in the operating room can be discussed and reflected upon in plenum, to benefit the overall section/department and thus also the surgical teams. We believe such an approach could create a bridge to overcome the difficulty surgeons have of appreciating the value of interpersonal skills in patient safety [38,39], i.e. in this paper the sharing/communication of insights across disciplines on how to combine different types of knowledge in surgery.

Another approach would be to include questions in the World Health Organization (WHO) Surgical Safety Checklist, on the types of knowledge used during a particular operation (i.e. does the team have knowledge from previous experiences with the particular type of operation that could aid safe work practices?). The checklist safety tool has increasingly been adopted worldwide and has also demonstrated reduction in the rates of death and complications during surgery [40,41]. We believe inclusion in the checklist could provide further benefits to surgery, by strengthening the individual and team awareness of knowledge elements and possibly also adaption to current surgical practices.

Finally, we suggest that insights into ways of combining knowledge should be embedded into the current medical and nursing educational curricula and training efforts, to further enhance safe work practices.

## Conclusions

The paper set out to explore and document the nature of the knowledge interdisciplinary teams use in surgical operations, in order to achieve safe work practices. What we found was that different elements of knowledge are combined to achieve safe work practices in surgical operations. We also found that these elements overlap with existing findings in health and medicine literature, while at the same time providing nuances of their own. We believe these nuances are an essential part of the repertoire operating teams need in their everyday practices, in order to move "beyond competence at needle insertion to incorporate unwritten strategies for increasing success" [[42], p. 405]. Thus, future research efforts should be used on exploring and documenting the relationships between various elements of knowledge and safe work practices, in different surgical settings and countries.

## Competing interests

The authors declare that they have no competing interests.

## Authors' contributions

SH was responsible for the study conception and design, drafting of the manuscript, data collection, data analysis, and critical revisions for important intellectual content. KA participated in the study conception and design, data analysis, and made critical revisions of the manuscript. JGH participated in the data collection, data analysis, and made critical revisions of the manuscript. All authors read and approved the final manuscript.
